# *ALTERED MERISTEM PROGRAM 1* Is involved in Development of Seed Dormancy in Arabidopsis

**DOI:** 10.1371/journal.pone.0020408

**Published:** 2011-05-26

**Authors:** Jayne Griffiths, Jose M. Barrero, Jennifer Taylor, Chris A. Helliwell, Frank Gubler

**Affiliations:** Division of Plant Industry, CSIRO, Canberra, Australian Capital Territory, Australia; University of Melbourne, Australia

## Abstract

Mutants in the rice *PLASTOCHRON 3* and maize *VIVIPAROUS 8* genes have been shown to have reduced dormancy and ABA levels. In this study we used several mutants in the orthologous gene *ALTERED MERISTEM PROGRAM 1* (*AMP1*) to determine its role in seed dormancy in Arabidopsis. Here we report that there are accession-specific effects of mutations in *AMP1*. In one accession, *amp1* mutants produce seeds with higher dormancy, while those in two other accessions produce seeds of lower dormancy. These accession-specific effects of mutating *AMP1* were shown to extend to ABA levels. We assayed global gene transcription differences in seeds of wild-type and mutant from two accessions demonstrating opposing phenotypes. The transcript changes observed indicate that the *amp1* mutation shifts the seed transcriptome from a dormant into an after-ripened state. Specific changes in gene expression in the mutants give insight into the direct and indirect effects that may be contributing to the opposing dormancy phenotypes observed, and reveal a role for *AMP1* in the acquisition and/or maintenance of seed dormancy in Arabidopsis.

## Introduction

Dormancy is an adaptive trait that is defined by the temporal inability of a seed to germinate under favourable conditions. Dormancy is initiated during seed development. The importance of the timing of seed germination in plant life cycles has resulted in a range of dormancy mechanisms to enhance survival in various environments. The mechanisms involved in blocking embryo growth in dormant seeds and the acquisition of embryo growth potential in non-dormant seeds are regulated by complex interactions between genetic and environmental factors. Understanding the general mechanisms controlling seed dormancy is important, as is uncovering species-specific factors that could facilitate the manipulation of seed dormancy in some plants, but not in others. These species-specific factors could be crucial for manipulating seed dormancy in different crops.

The model species Arabidopsis has long been used to study dormancy at the genetic and physiological levels [1]. These studies have revealed the importance of abscisic acid (ABA), gibberellins, light, temperature, nutrition and seed coat [2]. Genetic studies and more recent large scale studies investigating transcript changes, including transcription factor profiling [3]–[5], have highlighted the importance of ABA signalling in mediating loss of dormancy by after-ripening (a period of dry storage). Several studies in Arabidopsis and maize have also focused on the control of seed maturation and the acquisition of seed dormancy [6]. AFL (ABI3/FUS3/LEC2) B3 domain factors have been found to be critical in the seed maturation program. Mutations in these genes compromise desiccation tolerance, embryo identity and dormancy, and alter the regulation of, and the response to, important hormones in dormancy and germination such as ABA and gibberellins. One upstream factor regulating this class of genes is *LEAFY COTYLEDONS 1* (*LEC1*) [6]. Understanding how *LEC1* and other genes regulate the AFL B3 network could be very important for manipulating many important traits that arise during seed development such as seed dormancy.

The *VIVIPAROUS 8* (*VP8*) gene from maize has been shown to be a regulator of *LEC1* and the AFL B3 factors [7]. *vp8* mutant grains display low dormancy and show a viviparous phenotype. Mutations in the orthologous gene *PLASTOCHRON 3/GOLIATH* (*PLA3/GO*) in rice also produce viviparous grains [8]. In addition to loss of dormancy, mutations in *VP8* and *PLA3* produce plants with pleiotropic phenotypes including abnormal leaf morphology, altered shoot apical meristem organisation, shortened plastochron and abnormal hormone homeostasis. The viviparous phenotypes observed are correlated with reduced ABA content in rice seedlings and developing maize grains, and with increased expression of an ABA catabolic gene in maize [7], [8]. *VP8* and *PLA3* encode proteins related to mammalian glutamate carboxypeptidase IIs (GPCII) which process small peptides involved in metabolic and signalling pathways [9]. Members of this family are known to be involved in the removal of glutamates from neuropeptides and poly-gamma glutamated folate. The plant putative GPCIIs have a high degree of conservation with the animal GPCII's, possessing the N-terminal membrane spanning domain, conserved zinc residues and catalytic residues. However, the biochemical function of the plant GPCIIs is currently unknown.

The Arabidopsis orthologue of *VP8* and *PLA3* is *ALTERED MERISTEM PROGRAM 1* (*AMP1*) [10]–[11]. Mutations in *AMP1* also give rise to pleiotropic phenotypes including an altered number of cotyledons, de-etiolation in dark grown seedlings, increased leaf initiation, dwarfing, earlier flowering time and semi-sterility [10]. Whilst there are similarities in the phenotypes of *amp1*, *vp8* and *pla3* mutants, there are also some species specific effects. For example, Arabidopsis *amp1* mutants have been shown to have increased cytokinin content [10]. A similar phenotype was also found in rice *pla3* but at a lower extent [8]. No difference in cytokinin levels has been observed in maize *vp8* [7].

There have been a number of studies on the effect of *amp1* alleles on whole plant development [10], [11] and seedling [12], however, little research has been done into the seed phenotype of these mutants. In the present study we focus on the role of *AMP1* on seed dormancy and germination in Arabidopsis by analysing *amp1* mutant alleles in different accessions which display different levels of seed dormancy. We have analysed the ABA content and global gene expression changes in *amp1* mutant seeds to gain insight into the possible mechanism(s) by which *AMP1/VP8/PLA3* orthologues are influencing dormancy. We found that in Arabidopsis the *amp1* mutation has different effects on dormancy and on ABA levels in different accessions. More importantly we show that *AMP1* plays a conserved role during seed development in the acquisition or maintenance of seed dormancy, and that when mutated shifts the dormancy program into a germination-like program.

## Results

### Phenotypes of different *amp1* mutant alleles in Col-0, L*er* and C24 accessions

**Figure 1:**
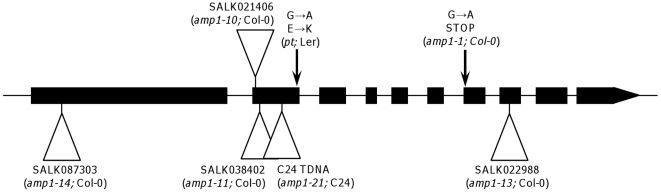
Positions of the mutations in *AMP1* used in this work. The structure of the *AMP1* gene is presented. Exons are shown as boxes, T-DNA insertion locations are indicated by triangles and the *amp1-1* and *pt* mutations by arrows.

**Figure 2:**
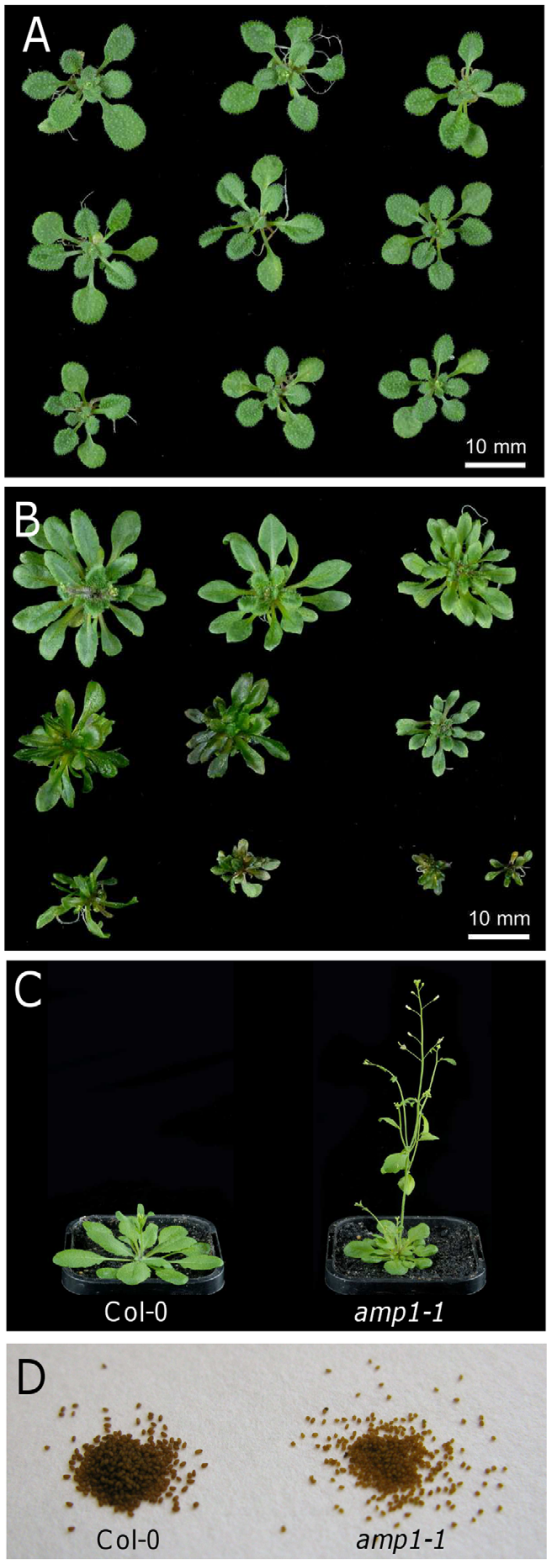
Phenotypes of *amp1-1*. Rosettes of Col-0 (A) and *amp1-1* (B) at 21 days after sowing. (C) Images of Col-0 and *amp1-1* 27 days after sowing. (D) Dry seeds from Col-0 and from *amp1-1*.

The effects of *AMP1* mutations on the vegetative and floral stages of the plant life cycle have already been reported [10], [12]. However, little is known about the effect of these mutations on seed dormancy. To determine the effect on seed dormancy we have studied several mutant alleles in different accessions. In the Col-0 background we studied the *amp1-1* mutation (encoding a premature stop codon) [11], three previously described knock-out T-DNA insertion mutants, *(amp1-10*, *-11*, and *-13*) [13] and one new knock-out insertion mutant (*amp1-14*) (Figure 1). The *primordia timing* allele (*pt*) [14] identified in L*er* and a new C24 allele (*amp1-21*, that carries a T-DNA insertion in the second exon at nucleotide 1268 from the translation start) were studied (Figure 1). All the mutations described here are recessive, and all mutant phenotypic traits are inherited in agreement with that. All the mutant plants displayed the same vegetative phenotype (Figure 2, Figure S1, S2 and S3), which has been described previously, such as an increased rate of leaf initiation (Figure 2B) and early flowering (Figure 2C). All the mutants studied showed complete penetrance but variable expressivity. Seeds produced by the mutant plants had a lighter pigmentation compared with their respective wild-types (Figure 2D).

**Figure 3:**
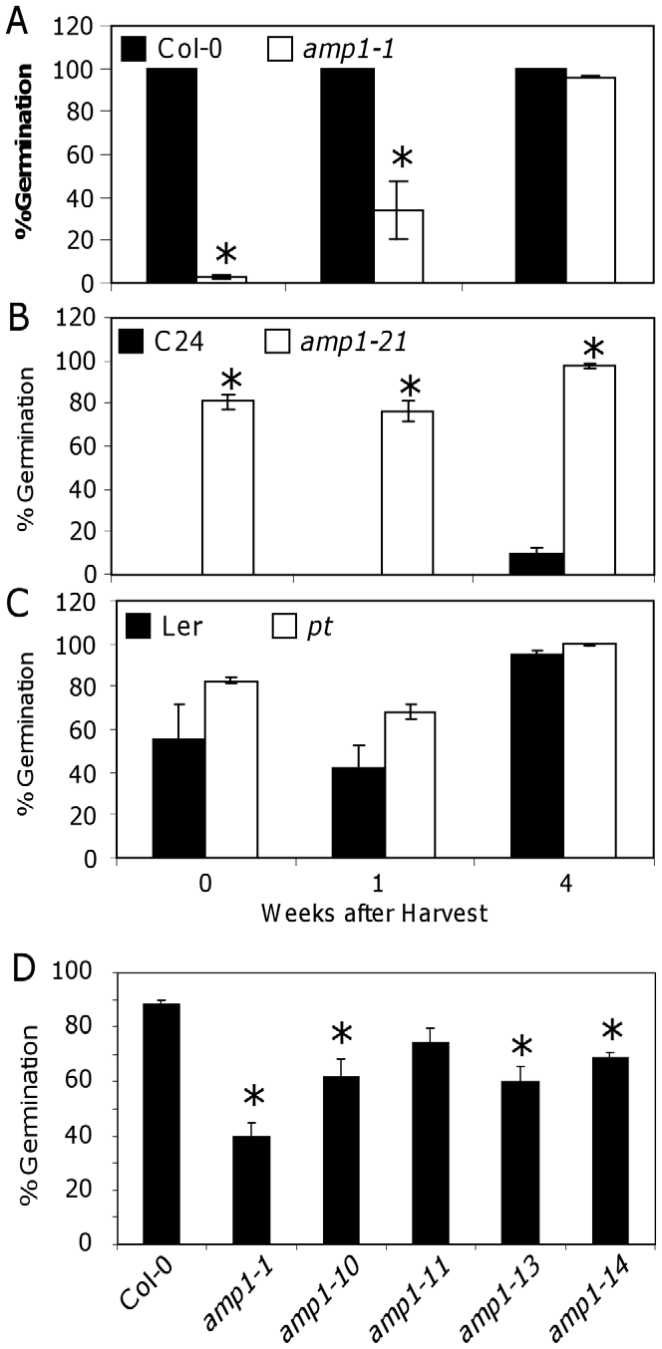
Dormancy phenotypes of *amp1* mutants. (A) % Germination seven days after sowing of bulk harvested Col-0 and *amp1-1* (A), C24 and *amp1-21* (B) and L*er* and *pt* (C) after-ripened for the indicated time. (D) Germination of all *amp1* mutant alleles in Col-0 background freshly-harvested seeds. Means of three biological replicates with their SE are shown. *Statistically significant difference in a Student's t-test (P<0.05; *n* = 3).

Mutations in *vp8* and *pla3* in maize and rice have been shown to produce plants with a viviparous phenotype [7], [8], similarly a mutant allele of *AMP1* has been reported to eliminate dormancy [15]. The nature of this mutation is not known. In order to characterise the effect of *amp1* mutations on dormancy, germination was measured in the Col-0, C24 and L*er* wild-types and in the *amp1-1*, *amp1-21* and *pt* mutants (all recessive mutations showing the same vegetative phenotypes) at 0, 1 and 4 weeks after harvest. Under our experimental conditions Col-0 seeds displayed almost no dormancy, with 100% germination from week 0. In contrast, *amp1-1* showed increased dormancy, with values of 5% germination at week 0, 30% at week 1 and around 100% at week 4 after harvest (Figure 3A). C24 seeds displayed strong dormancy, with 10% germination after 4 weeks of after-ripening. Surprisingly, *amp1-21* showed a dramatic dormancy loss, with 80% germination in week 0 after harvest (Figure 3B). L*er* showed an intermediate level of dormancy in our experimental conditions, having 50% germination at week 0 and around 90% at week 4 after harvesting. The *pt* mutant did not show a significant change in dormancy (Figure 3C).

In order to demonstrate that the increased dormancy phenotype of the *amp1-1* mutant was not an allele-specific effect, we also analysed the dormancy of the other four Col-0 mutants *amp1-10*, *-11*, *-13* and *-14*. These four T-DNA insertion mutants share the same vegetative phenotype as *amp1-1* (Figure S3) and also produced seeds that were more dormant (40–75% germination) than Col-0 (90% germination) after 7 days imbibition (Figure 3D). The germination rate of after-ripened seeds was also altered in the five *amp1* Col-0 mutants, with a requirement for a slightly longer imbibition time to reach 100% germination (data not shown).

### Gene expression analysis in wild-type and *amp1* mutant alleles

**Figure 4:**
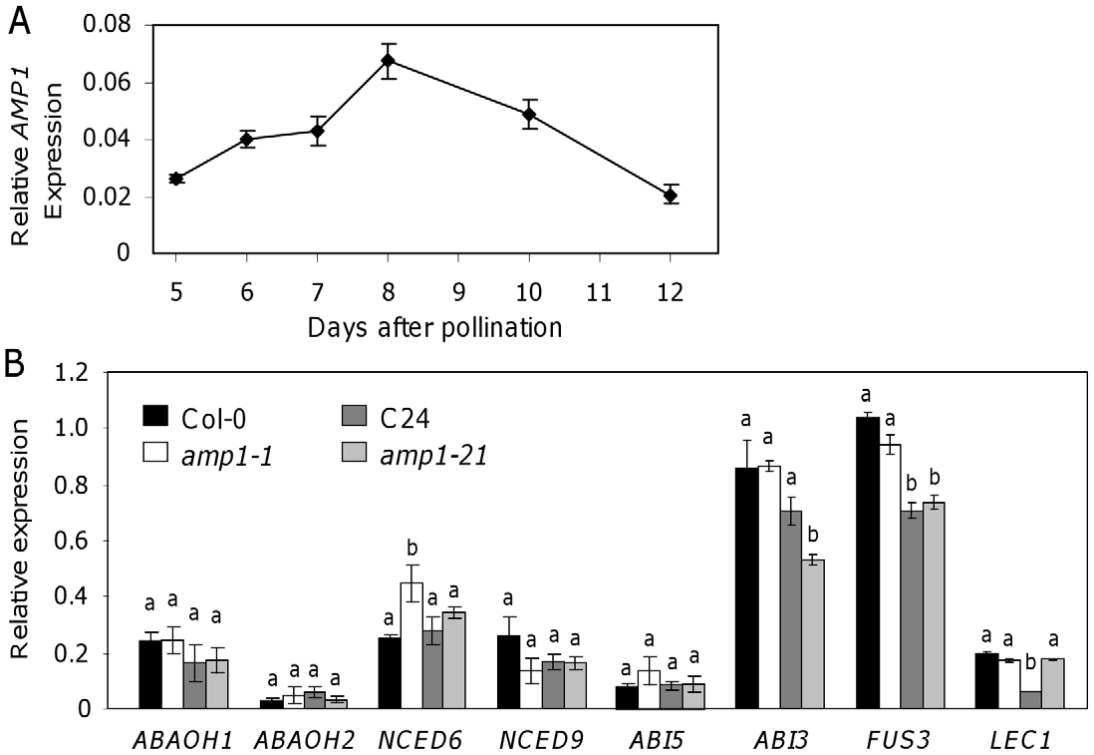
mRNA expression of *AMP1* and other genes involved in dormancy acquisition. (A) *AMP1* mRNA expression during seed development in Col-0. Under our experimental conditions seeds were considered mature at 14–15 days after pollination (B) Expression of several ABA-related genes and of *AFL* group of genes in Col-0, *amp1-1*, C24 and *amp1-21* developing seeds at 10 days after pollination. Means of three biological replicates with their SE are shown. Letters above bars identify statistically significant different values for each gene in a Student's t-test (P<0.05; *n* = 3).

The temporal expression pattern of *AMP1* was studied during seed development in Col-0 seeds. *AMP1* expression peaked at 8 days after pollination (DAP) and then began to decline as maturation occurred, suggesting a role during early-to-mid seed development (Figure 4A).

We studied the expression of genes with roles in seed development and ABA regulation in several *amp1* mutants. Gene expression analysis was carried out in developing seeds removed from the silique at 10 DAP, just after *AMP1* expression reached its maximum. We studied the expression of the AFL B3 factors (*LEC1*, *ABI3* and *FUS3*), and several ABA metabolic and signalling genes, the two ABA biosynthetic genes *NINE-CIS EPOXYCAROTENOID DIOXYGENASE 9* (*NCED9*) and *NCED6* [16], the two ABA catabolic genes *CYP707A1* and *A2* [17], [18] and the ABA signalling gene *ABA-INSENSITIVE 5* (*ABI5*) [19]. All these genes were expressed predominantly in developing and mature seeds. In general these genes were similarly expressed between wild-type and mutant seed (demonstrating they are at a similar developmental stage), with some minor differences (Figure 4B). *NCED6* was slightly up-regulated in *amp1-1* in comparison with Col-0. *ABI3* was slightly down-regulated in *amp1-21* in comparison with C24. *LEC1* was up-regulated in *amp1-21* with respect to C24. Interestingly we found that *ABI3*, *FUS3* and *LEC1* were down-regulated in C24 in comparison with Col-0. Although we could only detect a few changes driven by the *amp1* mutations common to both accessions, we identified several allele-specific and accession-specific changes.

### ABA quantification in wild-type and *amp1* mutant alleles

**Figure 5:**
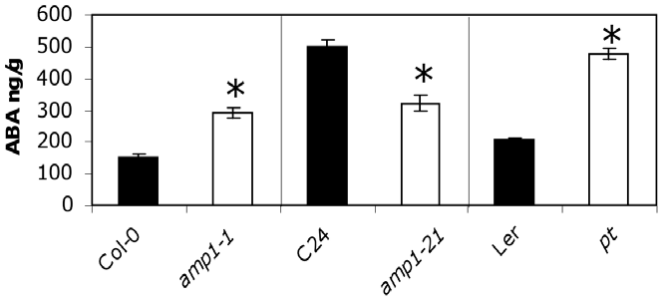
ABA content of dry wild-type and *amp1* mutant seeds. ABA was extracted from seeds one week after harvest. Means of three biological replicates with their SE are shown. *Statistically significant difference in a Student's t-test (P<0.05; *n* = 4).

In maize *vp8* and rice *pla3* mutants, a significant decrease in ABA levels in seeds and seedlings, respectively, was observed [7], [8]. In order to determine whether ABA levels were also changed in the different Arabidopsis *amp1* mutants, ABA levels were assayed. ABA levels were quantified in freshly harvested dry seeds from Col-0, C24, and L*er* accessions and from *amp1-1*, *amp1-21* and *pt* mutants. On a fresh weight basis *amp1-1* mutants contained significantly more ABA than Col-0 (p<0.01), *amp1-21* contained less ABA than C24, and *pt* contained more ABA than L*er* (Figure 5). For *amp1-1* and Col-0, ABA levels were also determined in developing seeds at 7, 10, 14 and 17 DAP, and an increase in ABA in *amp1-1* was found from 14 DAP to the dry seed state (data not shown). The levels of ABA in imbibed seeds were not quantified because the different germination rates of mutants and wild-type could affect the ABA measurements.

### Microarray analysis of *amp1-1*, Col-0, *amp1-21* and C24 developing seeds

To further analyse the effect of *AMP1* on dormancy, we compared the global gene expression changes produced by *amp1* mutations in two different accessions, Col-0 and C24. We chose these accessions because they displayed different dormancy levels: Col-0 produces seeds with very weak dormancy while C24 produced strongly dormant seeds. To identify gene expression differences that may confer the opposing dormancy phenotypes in Col-0 and C24 *amp1* knock-out mutants, microarray analyses were performed on Col-0 and *amp1-1* and on C24 and *amp1-21* developing seeds at 10 DAP.

Analysis of the data demonstrated that there were extensive changes in gene transcript levels in *amp1* mutants in both accessions, the majority of them probably being indirect effects. Genes that were differentially regulated in *amp1-1* and/or *amp1*-*21* compared to wild-type (>2-fold change in expression and an adjusted P-value<0.01) were selected (Table S1). In this group of 1597 genes, 863 were differentially regulated in *amp1*-*1* in comparison with Col-0 (Table S2), and 527 of them were specific to *amp1-1* and were not found in *amp1-21* (Table S5). In *amp1-21*, 1071 genes were differentially expressed in comparison with C24 (Table S3), and 734 of them were differentially regulated only in *amp1-21* but not in *amp1-1* (Table S6). Only 337 genes were commonly de-regulated in both mutants (Table S4). These 337 genes can be further split into two groups, those that were similarly regulated in both *amp1-1* and *amp1-21* (320 genes) and those that were regulated in opposing ways (17 genes).

The diversity of gene classes found was notable. GeneOntology (GO) annotation and TAGGIT re-annotation of the genes with germination/dormancy related terms [4] were used to determine whether there were any biological functions that were altered in opposing manners between the more dormant *amp1-1* and less dormant *amp1-21* and their wild-types, but we could not find any global change related with the phenotypes (Figures S4 and S5). However, it is notable that many genes found have functions in hormone synthesis and responsiveness. We did find several gene expression differences that could explain, at least in part, the mutant phenotypes. Table 1 shows selections of genes de-regulated in both mutants in the same manner, genes found only in *amp1-1* and genes found only in amp*1-21*. Of the selection of genes affected by *amp1-1*, At2g33830 is associated with bud dormancy and was up-regulated 20.4-fold; At2g16060 encodes the HEMOGLOBIN1 protein and was up-regulated 5.6-fold; *MYB118* was up-regulated 2.5-fold; and the gibberellin biosynthetic genes *KAO1* and *GA20OX2* were down-regulated −2.4 and −3.3-fold respectively. Among the genes affected by *amp1-21*, *CLAVATA 1* (*CLV1*) [20] was up-regulated 2.6-fold; *MYB51* and *MYB67* were down-regulated −2.5 and −4.5-fold, respectively; and *ABA3* was down-regulated −2.8-fold. There are several interesting genes that were up-regulated by both *amp1-1* and *amp1-21*, such as the meristematic gene *CLAVATA* 3 (*CLV3*) [20], *CYTOKININ RESPONSE FACTOR 2* (*CRF2*), and *CYP78A5*. There are also several genes that were down-regulated by both mutations, including *SCARECROW-LIKE 1* (*SCL1*) [21], *DELAY OF GERMINATION 1* (*DOG1*) [22], *TRANSPARENT TESTA 10* (*TT10*) [23], *GAST1 PROTEIN HOMOLOG 2* (*GASA2*) [24], *GASA3*, and *ETHYLENE RESPONSE FACTOR 72* (*ERF72*).

**Table 1:** Selected genes from the microarray analysis.

AGI	Annotation	*amp1-1* vs Col-0	*amp1-21* vs C24	adj.P.Val
*Genes de-regulated in both mutants (same regulation)*		**Fold Change**	**Fold Change**	
At2g27250	*CLV3 (CLAVATA3)*	4.1	8.0	2.71E-05
At4g23750	*CRF2 (CYTOKININ RESPONSE FACTOR 2)*	2.7	3.8	1.13E-03
At1g13710	*CYP78A5*	2.4	3.0	7.95E-05
At1g21450	*SCL1 (SCARECROW-LIKE 1)*	−2.1	−2.4	1.24E-02
At5g45830	*DOG1 (DELAY OF GERMINATION 1)*	−2.7	−2.6	2.05E-02
At5g48100	*TT10 (TRANSPARENT TESTA 10)*	−3.5	−2.6	1.44E-04
At4g09610	*GASA2 (GAST1 PROTEIN HOMOLOG 2)*	−4.8	−9.0	4.11E-04
At4g09600	*GASA3 (GAST1 PROTEIN HOMOLOG 3)*	−6.7	−8.8	6.14E-03
At3g16770	*ERF72 (ETHYLENE-RESPONSIVE)*	−20.1	−27.8	3.45E-05
*Genes only de-regulated in* amp1-1				
At2g33830	dormancy/auxin associated family protein	20.4	1.6	6.89E-03
At2g16060	*GLB1* (ARABIDOPSIS HEMOGLOBIN 1)	5.6	−1.5	3.91E-02
At3g27785	*MYB118*	2.5	1.1	1.19E-04
At1g05160	*KAO1* (ent-kaurenoic acid hydroxylase)	−2.3	−1.0	2.54E-03
At5g51810	*GA20OX2 (GIBBERELLIN 20 OXIDASE 2)*	−3.3	−1.8	2.05E-03
*Genes only de-regulated in* amp1-21				
At1g75820	*CLV1 (CLAVATA 1)*	1.5	2.6	1.08E-03
At1g17950	*MYB52*	−1.4	−2.5	6.23E-03
At1g16540	*ABA3 (ABA DEFICIENT 3)*	−1.2	−2.8	3.78E-02
At3g12720	*MYB67*	−1.8	−4.5	3.80E-02

Comparisons were carried out using developing seeds harvested at 10 days after pollination. Fold change was calculated dividing the expression in the mutant by the expression in the wild-type.

Given the opposite dormancy phenotypes of the *amp1-1* and *amp1-21* mutations, we have listed all 17 genes that were regulated in an opposing manner by the two mutations (Table 2), as these may contribute to the opposite phenotypes. Some of the genes from this list were expressed at different levels between the wild-types Col-0 and C24, as is the case of the ABA responsive gene *RESPONSIVE TO ABA 18* (*RAB18*) [25]. This gene was expressed more highly in C24 than in Col-0, however the *amp1-1* mutation increased the expression of this gene in Col-0, while the *amp1-21* mutation decreased it in C24. Another gene from this group was *EARLY LIGHT-INDUCED PROTEIN 1* (*ELIP1*) [26], which is implicated in light signalling, and was up-regulated in *amp1-21* and down-regulated in *amp1-1*.

**Table 2:** Genes de-regulated in both mutants in opposing manners.

AGI	Annotation	*amp1-1* vs Col-0	*amp1-21* vs C24	adj.P.Val
At5g38780	S-adenosyl-L-methionine:carboxyl methyltransferase	17.8	−2.1	1.27E-04
At3g20950	CYP705A32	6.5	−2.3	2.36E-04
At1g75030	ATLP-3 (THAUMATIN-LIKE PROTEIN 3)	3.3	−1.9	3.70E-02
At5g09570	Unknown	2.9	−3.3	3.41E-04
At2g23240	plant EC metallothionein-like family 15 protein	2.7	−2.1	1.19E-02
At5g48850	*SDI1 (SULPHUR DEFICIENCY-INDUCED 1)*	2.2	−11.0	5.21E-02
At5g49360	*BXL1 (BETA-XYLOSIDASE 1)*	2.1	−2.0	2.36E-02
At5g66400	*RAB18 (RESPONSIVE TO ABA 18)*	2.1	−2.8	7.58E-02
At5g57240	*ORP4C (OSBP-RELATED PROTEIN 4C)*	−2.1	2.7	1.84E-03
At5g54470	zinc finger (B-box type) family protein	−2.1	2.8	6.63E-04
At3g12955	auxin-responsive protein-related	−2.3	3.1	5.63E-03
At2g28900	*OEP16-1 (OUTER PLASTID ENVELOPE PROTEIN 16-1)*	−2.3	4.7	7.34E-06
At3g06360	*AGP27 (ARABINOGALACTAN PROTEIN 27)*	−2.5	2.1	2.97E-02
At2g17850	Unknown	−2.5	2.8	1.33E-02
At4g14980	Similar to DC1 domain containing protein	−2.7	10.3	6.49E-06
At3g22840	*ELIP1 (EARLY LIGHT-INDUCABLE PROTEIN)*	−2.8	2.0	1.45E-02
At1g43590	Transposable element gene	−2.8	2.0	1.56E-02

Comparisons were carried out using developing seeds harvested at 10 days after pollination. Fold change was calculated dividing the expression in the mutant by the expression in the wild-type.

**Figure 6:**
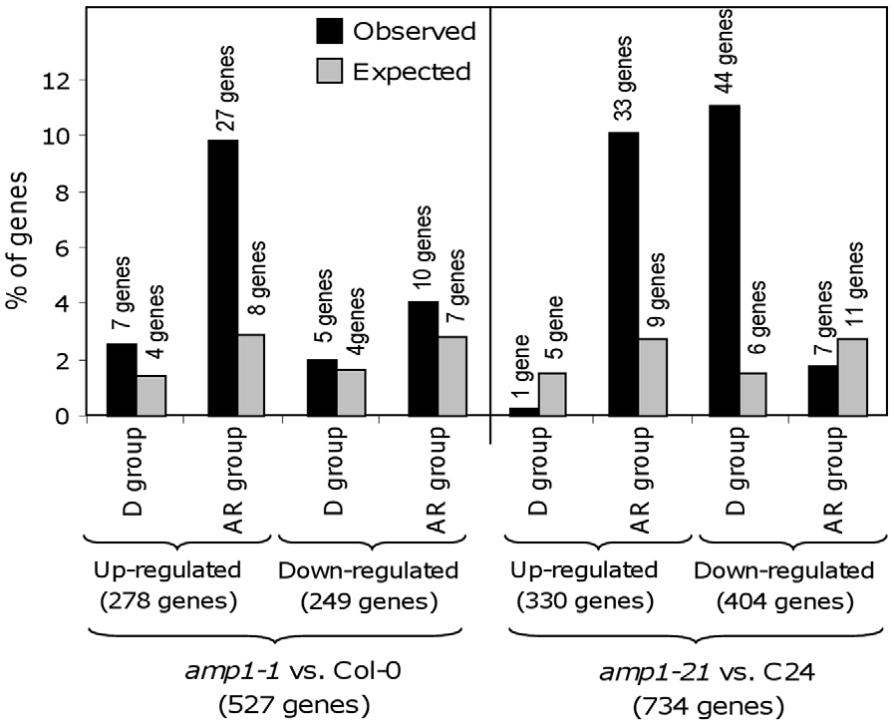
Analysis of the genes de-regulated in *amp1-1* and *amp1-21*. The percentage of genes observed and expected (considering random distribution) for each category is shown. The number of genes observed and expected is also indicated above each column. D (Dormant) and AR (After-Ripened) groups were defined in Cadman et al. (2006), containing 442 and 779 genes, respectively.

We analysed further the 527 genes specifically de-regulated in *amp1-1* (Table S3) and the 734 genes specifically de-regulated in *amp1-21* (Table S5). In a study of Cvi seeds [3], mRNA expression was analysed in several dormancy and after-ripening states, using microarrays. In that work, a core list of 442 genes was found to be more highly expressed in all dormant states analysed and 779 genes was highly expressed in all the after-ripened states. In C24, 330 genes were up-regulated by *amp1-21*, 10% of them were classified into the “after-ripened” group by Cadman et al. 2006 (only 2.5% expected by random distribution); 404 were down-regulated by *amp1-21* and again 11% (only 1.5% expected) of them were classified into the “dormant” group (Figure 6). However only 1% (more than 2.7% expected) of the down-regulated genes appear in the “after-ripened” set, and 0.3% (more than 1.5% expected) of the up-regulated genes appear in the “dormant” data set. In the case of Col-0, 278 genes were up-regulated by *amp1-1*, and again around 10% (only 2.9% expected) of them were classified into the “after-ripened” group and 2.5% (1.4% expected) in the “dormant”. Out of the 249 genes down-regulated by *amp1-1*, only 2% (1.6% expected) belonged to the “dormant” group and 4% (2.8% expected) to the “after-ripened” (Figure 6).

## Discussion

### Accession-specific effects of *amp1* mutations on seed dormancy

In this study we have investigated the effect on dormancy of the *AMP1* gene in Arabidopsis, using several recessive mutations in different accessions. The recessive nature of the *amp1* mutations and the similar phenotypes of alleles with insertions in different positions in the gene suggest these are lost-of-function mutant, although we cannot rule out the possibility that truncated peptides with altered activity are produced. Previously, *AMP1* was identified as a putative glutamate carboxypeptidase with similarities to the human N-acetylated alpha-linked acidic dipeptidase (NAALADase) proteins [11]. It has been suggested that there could be conservation of the biochemical function of this protein family across plants and animals, due to the conserved N-terminal membrane spanning domain, zinc binding and catalytic residues in both plant and mammalian GPCIIs [8], [11]. However the biochemical function of AMP1 has not been demonstrated. All *amp1*, *pla3* and *vp8* mutants display pleiotropic phenotypes. Whilst there are some similarities between the mutants, there are also significant differences. It has been shown that *pla3* and *vp8* are viviparous and there has been a previous report of a viviparous allele of *amp1* in Arabidopsis [15]. We show that whilst dormant C24 can be made non-dormant by the *amp1-21* mutation, non-dormant Col-0 can be made more dormant by mutating the same gene. We have studied five different Col-0 mutant alleles that are affected in different gene regions (Figure 1), all are recessive and show the same vegetative phenotypes, and all of them showed an increase in seed dormancy (Figure 3). It is unlikely that the different dormancy effects in Col-0 and C24 are caused by the differences between mutant alleles, because for example *amp1-11* and *amp1-21* are very similar alleles both carrying T-DNA insertions in the second exon. All these together strongly supports that mutation of *AMP1* produces accession-specific effects. This phenomenon has been reported before in regards to flowering time in Arabidopsis. The *pt* allele of *AMP1* in the L*er* accession has been shown to have no effect on flowering time, whereas mutants in a Col-0 background require fewer days to bolt than wild-type [27]. These differential dormancy and flowering time phenotypes suggest that genetic background can have a strong impact on the effect of mutations in *AMP1*. Accession-specific effects of mutations in *AMP1* orthologues have also been reported in maize, where the viviparous phenotype is observed in the SC background but not the W22 background, where it produces a defective embryo rather than vivipary [7].

Beside the accession-specific dormancy phenotype, we have found a common mutant trait in the seeds of *amp1-1*, *amp1-21* and *pt*. The seed coats of the mutant seeds are lighter in colour than the wild-type seed coats. Another trait, out of the scope of this manuscript, common to the three mutants is the highly variable phenotype expressivity in the vegetative tissues (Figure 2, Figure S1, S2 and S3).

### ABA-related effects of *amp1* mutations

ABA concentration increases during later stages of seed development, inducing seed dormancy and desiccation tolerance [28]. In the maize *vp8* mutant, the lack of dormancy is correlated with decreased ABA levels in the developing grain [7]. We have shown that in Arabidopsis, ABA levels in dry seeds are not clearly correlated with the level of dormancy observed in the seeds from three different mutants in three different wild-type accessions. However in the case of *amp1-1* and Col-0 and of *amp1-21* and C24, the level of ABA could be one of the reasons explaining the increase and the decrease in dormancy, respectively.

We analysed the expression level of some of the key genes involved in ABA synthesis, catabolism and signalling in the seed. We looked at the expression of *NCED6* and *NCED9* and saw a minor increase in the expression of *NCED6* only in *amp1-1*, and no change in *NCED9*. ABA catabolic genes are expressed throughout seed development, the most abundant being *CYP707A1* during the mid maturation stage and *CYP707A2* at late maturation [29]; we did not detect expression differences for these genes. Finally the expression of the ABA signalling-related genes *ABI3* and *ABI5* appears not to be effected in *amp1-1*, and only *ABI3* expression is slightly reduced in *amp1-21*. Therefore we could not find a consistent effect of the *amp1* mutations on ABA metabolism or sensitivity in different accessions, suggesting the differences in the ABA level may be due to indirect regulation, for example by feedback signals coming from changes in other hormones (a change in cytokinins and in gibberellins and ethylene signalling has been already reported in *amp1-1* [10], [12]).

We have also tested the putative role of *AMP1* in regulating the AFL B3 domain factors (ABI3/FUS3/LEC2). In maize and rice, *AMP1* orthologues have been shown to regulate the expression of this important set of genes during seed development [6]. We were able to validate that role in the accession C24, where *amp1-21* affected the expression of *ABI3* and *LEC1*. In contrast, in Col-0, *amp1-1* did not appear to affect the expression of any of the AFL genes.

### Specific gene expression changes in *amp1-1* and *amp1-21* mutants

We have grouped our microarray results into different categories: genes differentially regulated only in *amp1-1* or only in *amp1-21*, genes differentially regulated by both mutations in both accessions in the same manner (Table 1), and genes differentially regulated by both mutations but in opposing manners (Table 2). Those that are similarly regulated in *amp1-1* and *amp1-21* are likely to be specific to the *amp1* mutation rather than due to an effect of the background accession. Those regulated in one or the other accession will most likely be due to a background specific effect of the *amp1* mutation. And finally those differentially regulated in both accessions but in a different manner could be specific *AMP1* targets also regulated by accession-specific effects.

Among the genes that are similarly regulated in both Col-0 and C24 mutants are genes that have been previously identified as being involved in meristem regulation, such as *CLV3* [13]. This gene is up-regulated in both mutants, and although the increased meristem size of *amp1* mutants does not correlate well with the over-expression of *CLV3*, this could be compromising meristem development. Another gene that appears in this list is *CYP78A5*, which was previously identified in a microarray study of *amp1* mutant rosettes [11], suggesting it is specifically regulated by *AMP1* in at least two different tissues. We have highlighted a third gene up-regulated in both mutants, *CRF2*, that may be a consequence of the increased cytokinins in *amp1* previously described [10]. A gene that is expressed specifically in the developing seed (Arabidopsis eFP Browser [30], [31]) and that is down-regulated in both *amp1* mutants is *TT10*. TT10 is involved in the oxidation of procyanidins, which are clear in colour, to the brown oxidised procyanidins [23], [32]. Mutants in *TT10* have pale yellow seeds that darken gradually similar to *amp1* mutant seeds, suggesting that the down-regulation of *TT10* causes the colour phenotype. We also found several other genes down-regulated in *amp1-1* and *amp1-21* that could be related to seed dormancy and germination. Several of these are related to gibberellins; the *SCL1* transcription factor, a putative gibberellin regulator, and *GASA2* and *3*, two seed specific gibberellin-responsive genes [24]. Another interesting gene that is down-regulated more than 20-fold is ethylene-response factor *ERF72*; ethylene has been shown to promote germination and to be antagonistic to ABA [33]–[35] and it seems to act in the micropylar endosperm, a key seed tissue controlling germination [34]–[35]. A role of AMP1 in ethylene and GA signalling pathways during seedling development has already been described [12]. Finally we identified one of the better known dormancy-related genes, *DOG1* [22]. This gene reduces dormancy when mutated and is down-regulated in both *amp1-1* and *amp1-21* compared with wild-type seeds. This gene was first discovered as a QTL involved in seed dormancy using a Cvi x L*er* mapping population. The different *DOG1* alleles from Cvi and L*er* were correlated with high and low seed dormancy, respectively [22]. If C24 carries a *DOG1* allele that confers the high dormancy observed in C24 (similarly to the Cvi allele), its down-regulation by *amp1-21* would then explain the lack-of-dormancy phenotype. Conversely if Col-0 carries weak-dormancy *DOG1* allele (functionally similar to the L*er* allele), the down-regulation of this gene by *amp1-1* would not have any affect. Other explanations for the change in expression of *DOG1*, relate to changes in response to ABA, can be found. For example, the down-regulation of *DOG1* in the *amp1-21* mutant, which has reduced levels of ABA, could be related to the finding that the Cvi allele of *DOG1* is up-regulated by ABA [36].

From the group of genes de-regulated in *amp1-21* but not in *amp1-1*, we found a number that correlate well with the putative role of *AMP1* in other species. We also found another well characterised meristematic gene, *CLV1*, to be up-regulated. The ABA biosynthesis gene *ABA3* is down-regulated, consistent with the reduction in the levels of this hormone found in *amp1-21* seeds. Finally the transcription factors *MYB52* and *MYB67*, which were previously found to be up-regulated in dormant seeds [5], are significantly down-regulated in *amp1-21* again consistent with the after-ripened-like behaviour of this mutant.

In the Col-0 *amp1-1* mutant, we found several genes only de-regulated in this genotype that could explain the increase in dormancy produced by this mutation. At2g33830 encodes a dormancy/auxin associated family protein, previously shown to be involved in bud dormancy in several species. The mechanism controlling bud and seed dormancy is very similar and as this gene is up-regulated 20-fold in *amp1-1*, it is a good candidate to explain the change in dormancy observed in the mutant. *GLB1* is also very significantly up-regulated in *amp1-1*. This gene encodes the Arabidopsis HEMOGLOBIN 1 protein, which has been found to scavenge nitric oxide (NO) [37]. NO is a well known promoter of germination, and its depletion could lead to an increased dormancy [38]. A third gene up-regulated in *amp1-1* is the transcription factor *MYB118* that was found to be up-regulated in dormant seeds [5]. Interestingly two gibberellin biosynthetic genes, *KAO1* and *GA20OX2*, are down-regulated in *amp1-1*. The effect of gibberellins as ABA antagonists have been widely studied and a reduction in gibberellin levels could explain an increase in seed dormancy.

Genes de-regulated by both *amp1-1* and *amp1-21* in opposing manners could explain the opposite effect on seed dormancy caused by the two mutations. In Table 2 we have described all the genes belonging to this category, eight of them being up-regulated in *amp1-1* and nine of them in *amp1-21*. Many of the genes from this group have not been reported before to be related with dormancy or germination, but some are more than 10-fold differentially expressed in one mutant or the other. A number of these genes are also differentially expressed between the wild-types so could explain the differences in dormancy level between Col-0 and C24. From this group *RAB18*, an ABA-responsive factor commonly used as an ABA-response marker gene [25], is of particular interest. This gene is up-regulated in *amp1-1* and down-regulated in *amp1-21*, suggesting that the ABA pathway has been activated by the first mutation and repressed by the second, fitting with the phenotype of both mutants. Finally a second gene that may be important in the discussion of differences in dormancy is *ELIP1*, a light-induced gene described in many species [26], [39] and related with grain dormancy in barley [40]. In Arabidopsis, light is needed to promote germination and break dormancy. Interestingly in *amp1-1* the gene *ELIP1* is down-regulated as if the light activated pathway were repressed. In contrast, in *amp1-21*, *ELIP1* is up-regulated as would happen in a non-dormant seed. It is notable that the *amp1* mutation affects, directly or indirectly, two key elements in the control of dormancy and germination, the ABA and the light signalling pathways, in opposing manners in Col-0 and C24. These suggest that *AMP1* has a conserved role in dormancy acquisition during seed development, and could be used to investigate interactions between different dormancy regulatory pathways.

### Global gene expression changes in *amp1-1* or only by *amp1-21* mutants

Mutations in *AMP1* in C24 and Col-0 show some opposite phenotypes, suggesting that genetic factors in the background of the mutation are important. Microarray data generated from *amp1* mutations in the Col-0 and C24 backgrounds demonstrated that the functional classification of the de-regulated transcripts is largely similar. Genes regulated >2 fold in *amp1* mutants in Col-0 and C24 were compared to published data sets of dormant and after-ripened related genes in Cvi [3]. Very interestingly this analysis showed that the transcript signature of freshly harvested *amp1-21* seeds is mimicking the one of Cvi after-ripened seeds. The number of genes de-regulated in *amp1-21* found in the “dormant” or in the “after-ripened” sets was significantly different from the expected number of genes from a random distribution (Figure 6). Genes up-regulated in *amp1-21* were significantly enriched in genes from the “after-ripened” set from Cadman et al. (2006), and depleted in genes from the “dormant” set. Contrarily, genes down-regulated in *amp1-21* were significantly enriched in genes from the “dormant” set and depleted in genes from the “after-ripened” set (Figure 6). The germination phenotype observed in *amp1-21* correlates perfectly with these results. This effect of *amp1-21* turning C24 seeds into an “after-ripened-like” state is only partially observed in *amp1-1*. Again, the group of genes up-regulated by *amp1-1* is enriched in genes from the “after-ripened” set but is not depleted in genes from the “dormant” set. More importantly there is no significant over-representation of the “dormant” or “after-ripened” sets in the down-regulated genes (Figure 6).

This correlation between the transcript changes found in C24 vs. *amp1-21* and between the “dormant” and “after-ripened” states in Cvi, suggests that the *amp1* mutation is affecting specific pathways regulating dormancy. This supports the hypothesis that *AMP1* and orthologues in maize and rice are controlling a similar seed development mechanism involved in the acquisition of seed dormancy. However, mutation of *AMP1* in Col-0 appears to be affecting genes in a non-phenotype specific manner. An increase in the ‘after-ripened” group of genes is seen in the up-regulated set, but not in the down-regulated set. A hypothesis for that could be a dormancy model where Col-0 would produce seeds with a very weak dormancy because the dormancy mechanism existing in Cvi and C24 is absent in Col-0. In fact, more than 50% of the “dormant” set of genes that are down-regulated by the *amp1-21* mutation in comparison with C24 are also down-regulated in Col-0 wild-type in comparison with C24.

From these results we speculate that the some effects of the *amp1-21* mutation in eliminating dormancy in C24 may be conserved and direct, affecting the expression of dormancy-related genes like *DOG1* and affecting ABA and light sensitivity. The effect of *amp1-1* increasing dormancy in Col-0 could be a side effect resulting from the initial lack of deep dormancy in this accession and of fortuitous gene expression changes that partially restore dormancy levels. This is also in agreement with the effect of the *pt* mutation in L*er*, another low-dormancy accession. The *pt* mutant has increased levels of ABA in seeds, but its dormancy remained very similar to wild-type. In conclusion we propose a role for *AMP1* in the acquisition and/or maintenance of seed dormancy in Arabidopsis.

## Materials and Methods

### Plant lines and growth conditions

To generate seeds for dormancy and germination rate experiments, 10 Arabidopsis plants were grown in an 11 cm pot in soil (Debco seed raising mix +2 g litre^−1^ osmocote). Plants were grown in controlled environment cabinets set at a 16 hour photoperiod, with a light intensity of 180 µmol m^−2^ s^−1^ at a temperature of 20°C. Seeds were harvested in bulk 1 week after flowering had ceased when the plant was completely dry.

*amp1-1* and *amp1-21* were donated by Dr. Chris Helliwell. T-DNA mutants were identified using the Salk T-DNA insertion database and seeds were obtained from the Arabidopsis Biological Resource Center (http://biosci.ohio-state.edu/pcmb/Facilities/abrc/abrchome.htm). Homozygous mutant plants were then generated and dormancy and germination rate tested under our standard conditions.

Developing seeds were obtained from plants grown under the same conditions.

### Germination assays

For dormancy experiments involving C24, approximately 100 seeds were placed in 85 mm Petri dishes with 3 ml of water and three Whatman No. 1 70 mm diameter filter papers (Whatman International, Maidstone, England), as previously described [41]. Plates were then sealed with Parafilm and incubated at 20°C under continuous fluorescent light (100 µmol m-2 s-1) for 7 days. Germination was scored as the emergence of the radicle outside the seed coat and the endosperm. For dormancy experiments involving the Col-0 accession we found that the filter paper reduced dormancy, and therefore experiments were performed on 3 ml of 0.6% agarose in 35 mm Petri dishes [35]. Experiments were performed in triplicate for each line examined. All the seed used in an experiment were harvested from the same batch of plants, which were grown together at the same time and in the same environment.

### ABA quantification

The ABA content was measured from developing seeds and matured seeds. Between 100 and 300 developing seeds were extracted from siliques and transferred to pre-weighed foil boats on dry ice, seeds were then weighed. For the mature seed 50 mg of dry seed was used for the extraction. ABA for both developing and mature seed was extracted from quadruplicate replicates as described previously [42]. The ABA measurements were carried out using a Phytodetek Competitive ELISA kit (Agdia) following the manufacturer's protocol.

### Microarray and Quantitative real time PCR

Developing seeds for microarray and QRT-PCR experiments were removed from siliques at intervals after pollination and flash-frozen in liquid nitrogen. Total RNA was isolated from those developing seeds using the Qiagen RNA easy Plant mini kit (Qiagen), with Plant RNA isolation AID (Ambion) added to the extraction buffer. Isolated RNA was then treated with TurboDNAse (Ambion). The quality of RNA intended for microarrays was assessed on a 2100 Bioanalyzer (Agilent Technologies, Santa Clara, CA, USA) before being sent to Australian Genome Research Facility Ltd. (www.agrf.org.au; Melbourne, Victoria, Australia) for labelling and hybridisation to the Affymetix ATH1 genome array.

For transcriptome analysis, total RNA was isolated from 10 DAP developing seeds (for *amp1-1* around 20 siliques were dissected per replicate, and for wild-type around 10 siliques were dissected per replicate) from three independent biological replicates. Resulting microarray data was analysed using the limma package from Bioconductor (http://www.bioconductor.org/) in R (R Development Core Team, 2008). Microarray data are available in the ArrayExpress database (www.ebi.ac.uk/arrayexpress) under accession number E-MEXP-3181, following MIAME recommendations.

For QRT-PCR 0.5 µg of RNA was used to synthesise cDNA in a 20 µl reaction using SuperScript II (Invitrogen Life Sciences; http://www.invitrogen.com) using the protocols supplied. cDNA was diluted 25- fold and 10 µl of this was used in a 20 µl PCR reactions with Platinum Taq and SYBR Green (Invitrogen). Specific primers were designed, and are listed in Table S7. Reactions were run on a RG-3000A real-time PCR machine (Corbett Research; http://www.corbettlifescience.com) and data were analysed with Rotor-Gene software using the comparative quantification tool. The expression of *Cyclophillin* (At2g29960) was used as a control gene [41].

## Supporting Information

Figure S1 **Phenotypes of** ***amp1-21*** **.** Rosettes of C24 (A) *and amp1-21* (B) at 21 days after sowing.
(TIF)

Figure S2 **Phenotypes of** ***pt*** **.** Rosettes of L*er* (A) and *pt* (B) at 21 days after sowing.
(TIF)

Figure S3 **Phenotypes of all Col-0** ***amp1*** **mutant alleles studied in this work.** Rosettes of Col-0 (A), *amp1-1* (B), *amp1-10* (C), *amp1-11* (D), *amp1-13* (E) and *amp1-14* (F) at 21 days after sowing.
(TIF)

Figure S4 **GO classification of the genes differentially expressed in the microarray experiment.** Classification of genes expressed highly in Col-0 than in *amp1-1* (Col-0>amp1-1), *amp1-1*>Col-0, C24>*amp1-21* or *amp1-21*>C24 using the Gene Ontology (GO) molecular function. The proportional representation of the total gene set is shown.
(TIF)

Figure S5 **Classification of the genes differentially expressed from the microarray experiment using TAGGIT annotation.** Classification of genes expressed more highly in Col-0 than in *amp1-1* (Col-0>*amp1-1*), *amp1-1*>Col-0, C24>*amp1-21* or *amp1-21*>C24 using TAGGIT annotation [4].
(TIF)

Table S1 Probes differentially expressed in *amp1-1* and/or in *amp1-21*.
(XLS)

Table S2 Probes differentially expressed in *amp1-1*.
(XLS)

Table S3 Probes differentially expressed in *amp1-21*.
(XLS)

Table S4 Probes differentially expressed in both *amp1-1* and *amp1-21*.
(XLS)

Table S5 Probes differentially expressed only in *amp1-1*.
(XLS)

Table S6 Probes differentially expressed only in *amp1-21*.
(XLS)

Table S7 Primers used in this work.
(XLS)
