# Supplementary figures and images for: ALTERED MERISTEM PROGRAM 1 Is involved in Development of Seed Dormancy in Arabidopsis
Source: PLoS One. 2011 May 26;6(5):e20408. doi: 10.1371/journal.pone.0020408 (PMC3102729; doi:10.1371/journal.pone.0020408)

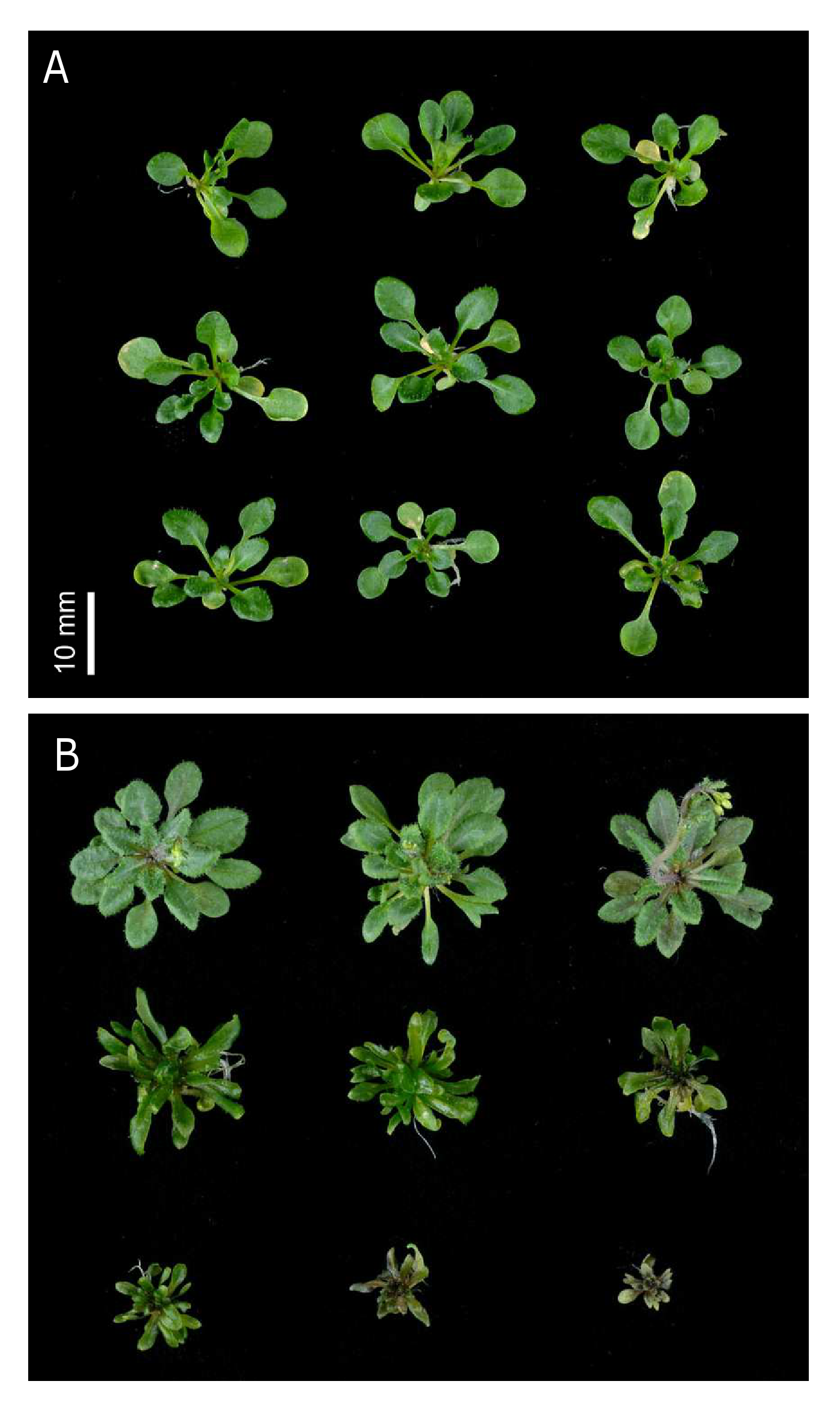

Supplement: Figure S1 — Phenotypes of amp1-21 . Rosettes of C24 (A) and amp1-21 (B) at 21 days after sowing. (TIF) [file pone.0020408.s001.tif]

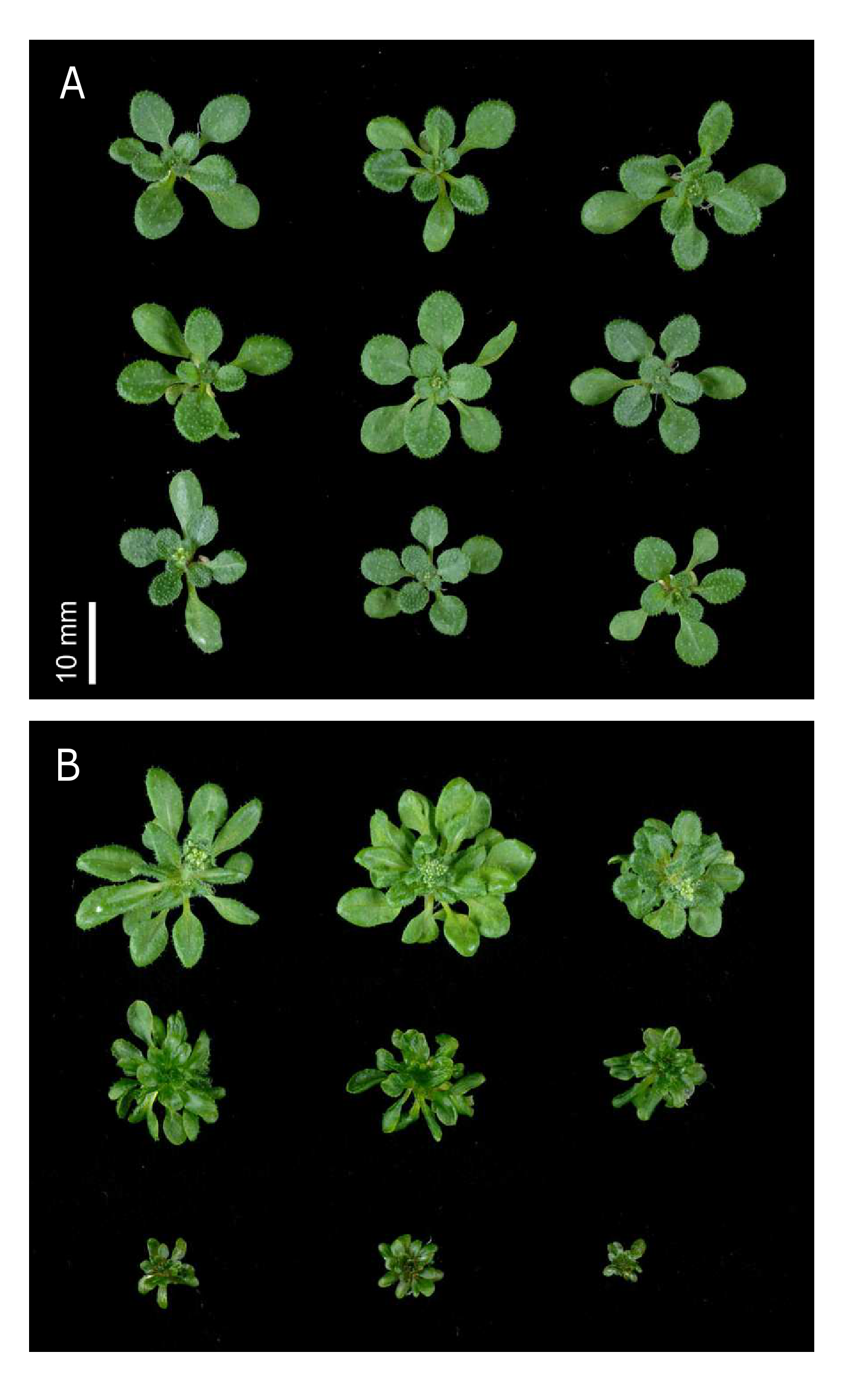

Supplement: Figure S2 — Phenotypes of pt . Rosettes of Ler (A) and pt (B) at 21 days after sowing. (TIF) [file pone.0020408.s002.tif]

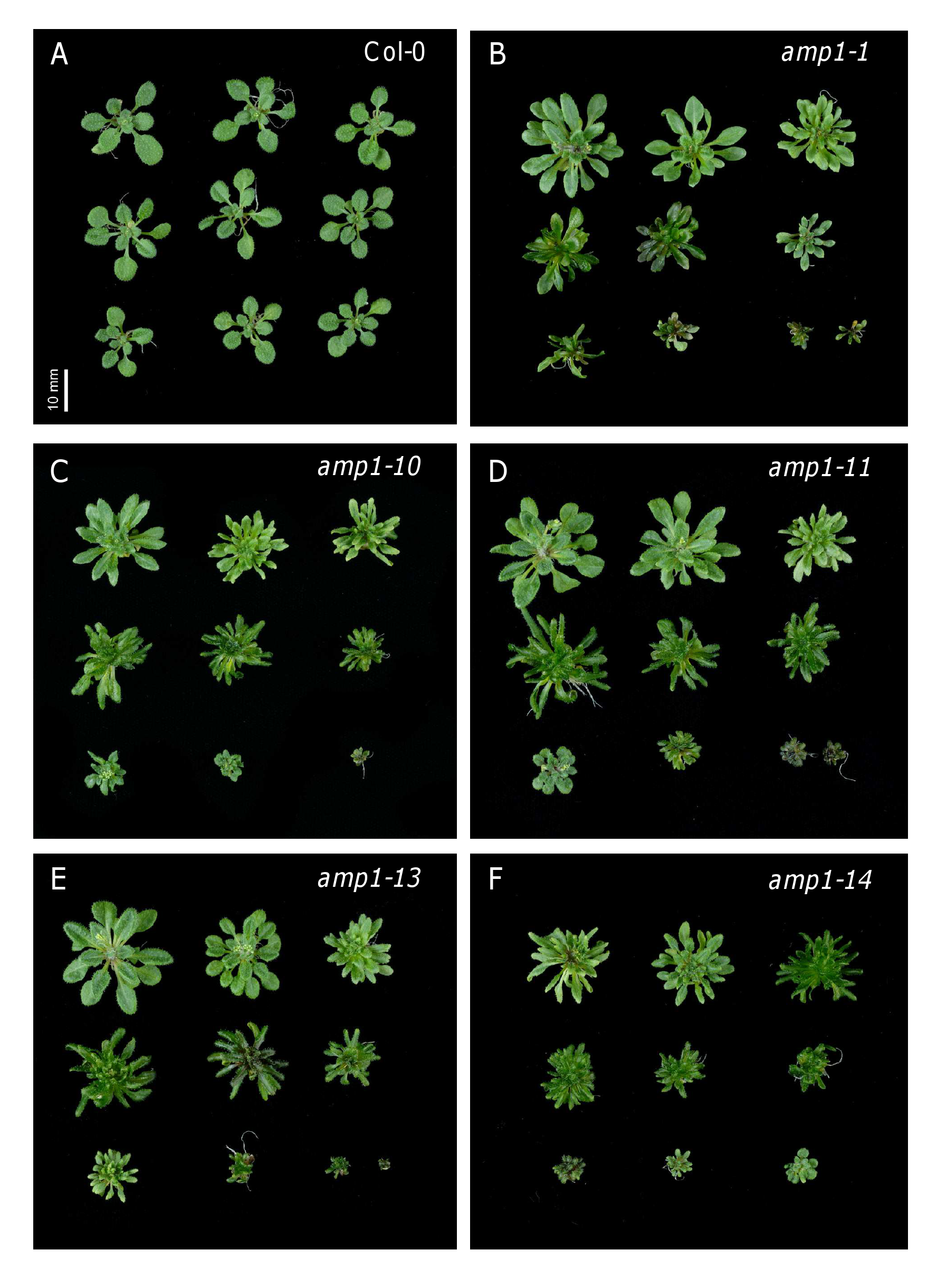

Supplement: Figure S3 — Phenotypes of all Col-0 amp1 mutant alleles studied in this work. Rosettes of Col-0 (A), amp1-1 (B), amp1-10 (C), amp1-11 (D), amp1-13 (E) and amp1-14 (F) at 21 days after sowing. (TIF) [file pone.0020408.s003.tif]

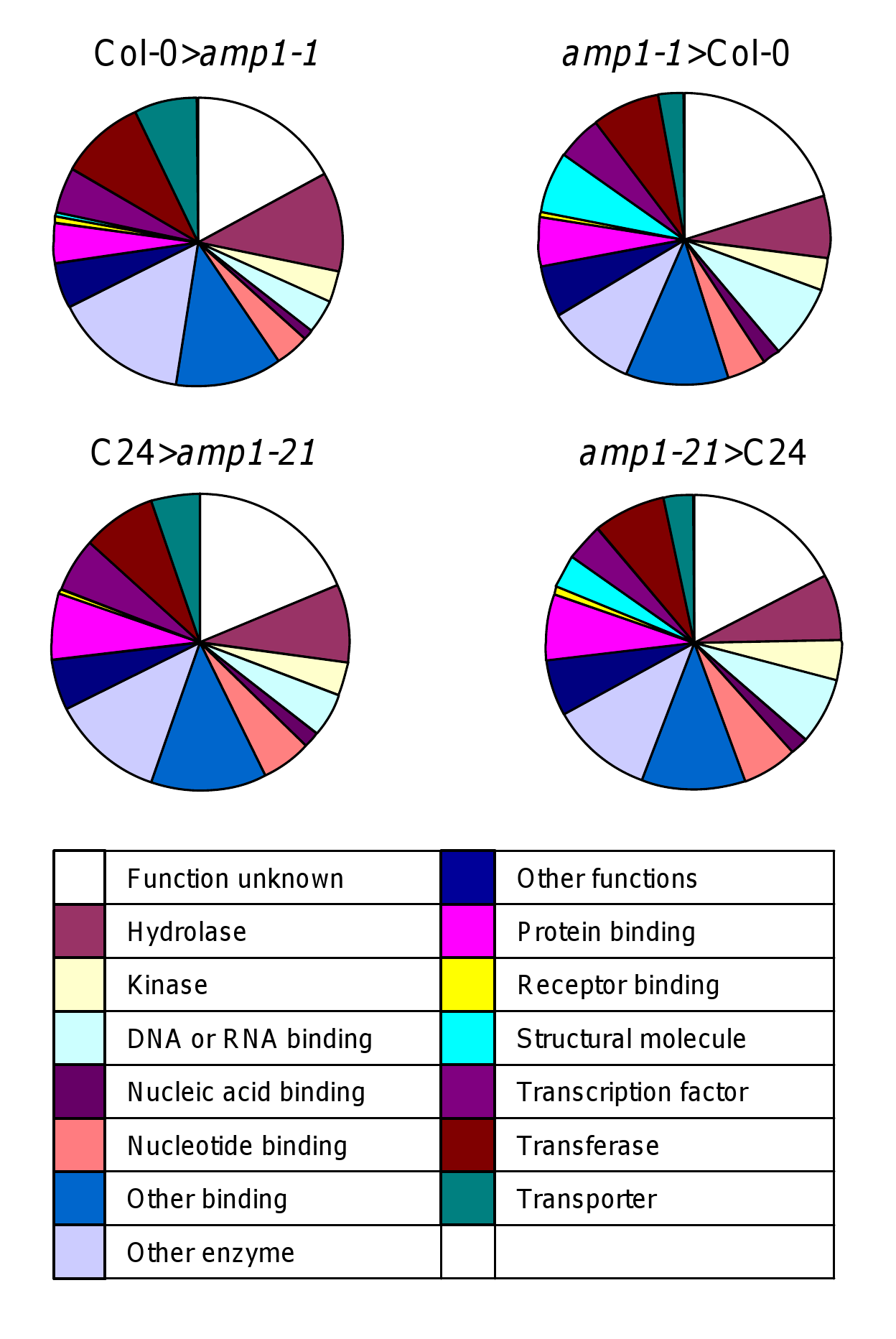

Supplement: Figure S4 — GO classification of the genes differentially expressed in the microarray experiment. Classification of genes expressed highly in Col-0 than in amp1-1 (Col-0>amp1-1), amp1-1>Col-0, C24>amp1-21 or amp1-21>C24 using the Gene Ontology (GO) molecular function. The proportional representation of the total gene set is shown. (TIF) [file pone.0020408.s004.tif]

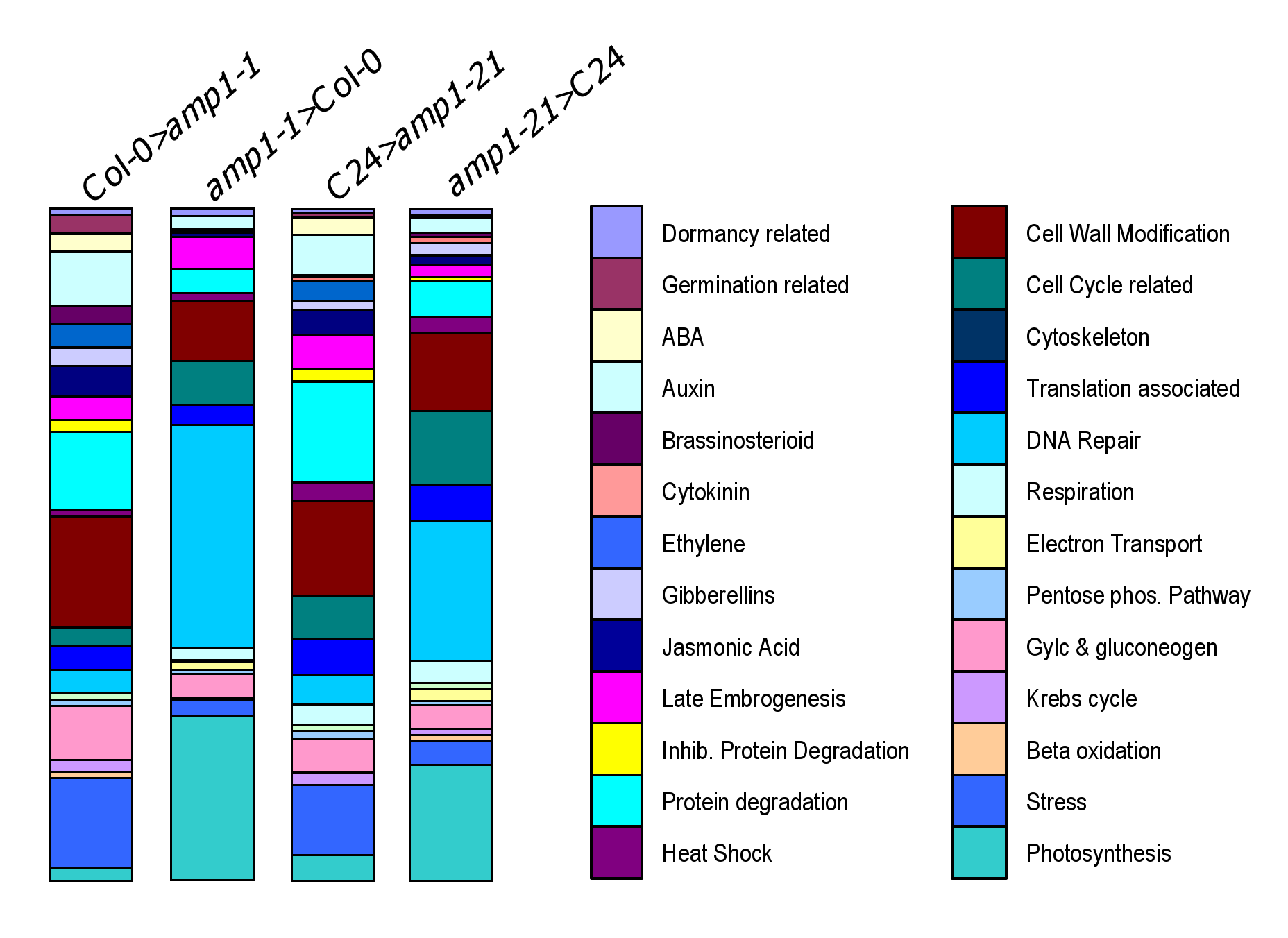

Supplement: Figure S5 — Classification of the genes differentially expressed from the microarray experiment using TAGGIT annotation. Classification of genes expressed more highly in Col-0 than in amp1-1 (Col-0>amp1-1), amp1-1>Col-0, C24>amp1-21 or amp1-21>C24 using TAGGIT annotation [4]. (TIF) [file pone.0020408.s005.tif]
